# Behavioral Experience-Sampling Methods in Neuroimaging Studies With Movie and Narrative Stimuli

**DOI:** 10.3389/fnhum.2022.813684

**Published:** 2022-01-27

**Authors:** Iiro P. Jääskeläinen, Jyrki Ahveninen, Vasily Klucharev, Anna N. Shestakova, Jonathan Levy

**Affiliations:** ^1^Brain and Mind Laboratory, Department of Neuroscience and Biomedical Engineering, Aalto University School of Science, Espoo, Finland; ^2^International Laboratory of Social Neurobiology, Institute of Cognitive Neuroscience, National Research University Higher School of Economics, Moscow, Russia; ^3^Massachusetts General Hospital, Massachusetts Institute of Technology and Harvard Medical School, Athinoula A. Martinos Center for Biomedical Imaging, Charlestown, MA, United States; ^4^Baruch Ivcher School of Psychology, Interdisciplinary Center Herzliya, Reichman University, Herzliya, Israel

**Keywords:** naturalistic stimuli, memory, attention, emotion, social cognition, language, movies, narratives

## Abstract

Movies and narratives are increasingly utilized as stimuli in functional magnetic resonance imaging (fMRI), magnetoencephalography (MEG), and electroencephalography (EEG) studies. Emotional reactions of subjects, what they pay attention to, what they memorize, and their cognitive interpretations are all examples of inner experiences that can differ between subjects during watching of movies and listening to narratives inside the scanner. Here, we review literature indicating that behavioral measures of inner experiences play an integral role in this new research paradigm *via* guiding neuroimaging analysis. We review behavioral methods that have been developed to sample inner experiences during watching of movies and listening to narratives. We also review approaches that allow for joint analyses of the behaviorally sampled inner experiences and neuroimaging data. We suggest that building neurophenomenological frameworks holds potential for solving the interrelationships between inner experiences and their neural underpinnings. Finally, we tentatively suggest that recent developments in machine learning approaches may pave way for inferring different classes of inner experiences directly from the neuroimaging data, thus potentially complementing the behavioral self-reports.

## Introduction

**FIGURE 1:**
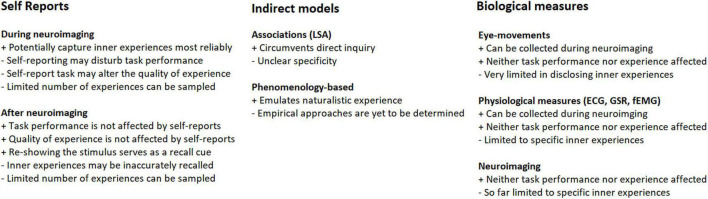
Summary illustration of the different types of approaches that are available for sampling of inner experiences. The potential strengths and limitations are indicated with + and – signs.

Movies and narratives are increasingly used as stimuli in functional magnetic resonance imaging (fMRI), electroencephalography (EEG), and magnetoencephalography (MEG) studies (Bartels and Zeki, 2004; Hasson et al., 2004; Dmochowski et al., 2012; Lankinen et al., 2014) (for a recent review, see Jaaskelainen et al., 2021). Such media-based stimuli are especially advantageous in that they allow for elicitation of a range of ecologically valid emotional and cognitive states in experimental subjects that would be difficult to achieve using traditional neuroimaging paradigms. This opens up possibilities to study the underlying neural mechanisms, which is one of the key questions in cognitive neuroscience. Elicitation of genuine strong emotions provides one good example where movies and narratives are more robust than other types of stimuli (Westermann et al., 1996). It is, however, highly important to take into account inter-subject variability in how the media-based stimuli are experienced. For example, a movie clip that is positive-emotional for one subject can be negatively experienced by another. Similarly, cognitive states and memories evoked by a narrative can significantly differ between subjects. Inter-subject variability in the elicited emotional and cognitive states can be utilized in the analysis of their neural basis. For example, if mental imagery is elicited only in some subjects during listening to an audiobook and if only those subjects exhibit activity in visual cortical areas, one can infer that visual cortical areas are important for mental imagery. It is worth mentioning that throughout the history of experimental psychology, there have been two competing schools of thought arguing on whether or not inner experiences can be consciously and reliably accessed by experimental subjects. For a recent account of this debate, and for arguments in favor of the validity of self-reporting subjective experiences see Hurlburt et al. (2017). A host of methods have been developed to assess the variability in behavioral experiences while subjects are watching movies or listening to narratives, and utilize the variability to guide neuroimaging data analyses (see Figure 1). Here, we review some of these important approaches.

## Individual Variability in Memory Encoding Guiding Neuroimaging Data Analysis

Post-experiment questionnaires can be used to quantify which aspects of movies or narratives have been memorized during neuroimaging. These provide a good example of a way to quantify the variability in how movies or narratives are experienced. The information provided by such questionnaires can then be used to guide neuroimaging data analyses. In one study, higher inter-subject correlation (ISC) of hippocampal hemodynamic activity, determined by calculating voxel-wise correlations between all pairs of subjects (Hasson et al., 2004; Kauppi et al., 2010; Pajula et al., 2012), went hand-in-hand with memorizability of movie contents 3 weeks later (Hasson et al., 2008). In another study, ISC of EEG activity correlated with subsequent memorization of movie clip content (Cohen and Parra, 2016). In a third example of this approach, subjects watched emotionally aversive vs. neutral movie clips during positron emission tomography. Memorization of events in the clips 3 weeks after the scan correlated with activity levels in the amygdala and orbitofrontal areas (Cahill et al., 1996). Naturally, assessing what is memorized of a stimulus afterward is straightforward to accomplish with post-neuroimaging questionnaires or recounts. Assessing how the subjects emotionally experienced media-based stimuli during neuroimaging is already a bit trickier. We review these approaches in the following.

## Assessing Emotional Experiences Elicited by Movies and Narratives After Neuroimaging

Movies and narratives represent a powerful tool for neuroimaging studies of emotions *via* their capability to induce robust emotions. Since emotional experiences can be highly variable across individual subjects, we argue that it is essential to ask the subjects about their experiences to inform the fMRI data analysis. The simplest approach is to ask the subjects afterward (Jones and Fox, 1992; Cahill et al., 1996; Aftanas et al., 1998; Jaaskelainen et al., 2008; Nummenmaa et al., 2012, 2014; Raz et al., 2012). In one study, subjects were asked to rate their experienced emotional valence and arousal continuously in two separate re-showings of the movie clips after the neuroimaging session. The ratings were then used to inform the neuroimaging data analyses. ISC of brain activity in default-mode and dorsal attentional networks were observed to co-vary with experienced valance and arousal, respectively (Nummenmaa et al., 2012). Naturally, obtaining continuous ratings for more than two classes of emotional experiences would be highly taxing for subjects, and obtaining even just one continuous rating is strenuous in case the movie or narrative is a long one. To circumvent this problem, we advise to obtain ratings of experienced categorical emotions as non-continuous variables (Jones and Fox, 1992).

In addition to emotional experiences *per se* one can obtain self-reports of specific aspects of emotional processing such as emotion regulation. Success in emotion regulation during watching of anger-eliciting movie clips has been observed to correlate with greater involvement of VMPFC in emotion regulation network (Jacob et al., 2018). Frontal EEG activity has been associated with better emotion regulation during induction of emotions (Nitschke et al., 2004; Dennis and Solomon, 2010). Degree of self-reported suspense has also been successfully used in fMRI data analyses (Metz-Lutz et al., 2010; Hsu et al., 2014). As a word of caution, the chances for obtaining reliable ratings are lower for more ambiguous and interpretable emotion categories (e.g., social emotions). We advise careful attention to be paid to how subjects are instructed, and running pilot experiments when asking to self-report categories of higher ambiguity.

In case of experienced humorousness, sadness, emotional valence, and emotional arousal, post-neuroimaging recall closely matches what is experienced during first-time watching of movie clips during neuroimaging. This is true at least when subjects are re-viewing the movie clips as memory cues for recall of what they had experienced during the first viewing of the clips in the scanner (Hutcherson et al., 2005; Raz et al., 2012; Jaaskelainen et al., 2016). Further studies are needed to determine for which emotional and other experiences the post-neuroimaging self-reports are accurate enough [e.g., using our published method (Jaaskelainen et al., 2016)]. In cases where post-scan emotion-ratings do not seem reliable, there are ways to sample emotional experiences during neuroimaging. These are briefly discussed below.

## Assessing Emotional Experiences Elicited by Movies and Narratives During Neuroimaging

In addition to the post-scanning self-reports of emotional experiences, paradigms have been developed where subjects continuously rate their experienced emotions while viewing the emotional movie clips in the scanner (Hutcherson et al., 2005). This approach potentially captures the emotional experiences more accurately than recall-based self-reporting, as the experiences might be different when viewing the emotional clips for the first vs. second time. Lending support for this concern, at least in case of some inner experiences there seem to be mismatches between retrospective reports and moment-to-moment experiences obtained in the scanner (Hurlburt et al., 2015). On the other hand, emotional responses might be altered by the ongoing rating task due to differential attentional demands.

There are findings showing that brain activity patterns elicited during passive viewing of emotional movie clips differ from brain activity patterns elicited while subjects are rating their emotions during viewing of the clips in the fMRI scanner (Hutcherson et al., 2005). Given this, we recommend quantification of emotional experiences *via* asking the subjects after the neuroimaging session how they had experienced the media-based stimuli while in the scanner. We advise to use the stimuli as recall cues when obtaining the ratings and validation of the post-scan ratings with separate behavioral experiments (Jaaskelainen et al., 2016). In addition to emotions, there are cognitive states, such as mental imagery or mentalization, that are elicited by media-based stimuli. In the following, we review behavioral methods with which such inner experiences can be quantified.

## Sampling Inner Experiences Other Than Emotions

In addition to experienced emotions, there are methods that have been developed to assess other inner experiences during neuroimaging such as mental imagery elicited by movies and narratives. These are called experience-sampling methods. One recent experience-sampling approach involved re-playing a narrative in segments to subjects after the neuroimaging session. The subjects were then tasked to produce a list of words in 20–30 s after each segment best describing what had been on their minds while they had heard the segment in the fMRI scanner. Word-list similarities were then estimated with latent semantic analysis (LSA). This was followed by representational similarity analysis (RSA; Kriegeskorte et al., 2008) of shared similarities between word-listings and voxel-wise fMRI activity as assessed by ISC metrics (Saalasti et al., 2019). Naturally, such word lists could also be probed for, e.g., occurrence of emotional words to estimate emotional reactions. Multi-dimensional scaling provides a manual alternative for automated algorithms such as LSA. In this approach, volunteers rate pair-wise similarities between word lists using, e.g., visual-analog scale (Bisanz et al., 1978).

There are also studies that have used experience sampling with other types of task paradigms. One example of such is a study where experimental subjects were presented intermittently with beeps during recording of resting-state fMRI. The beeps prompted the subjects to describe their thoughts and feelings just before each beep *via* answering specific questions (Hurlburt et al., 2015). In another study, it was observed that differences in inner experiences as sampled with post-scanning self-reports predicted differences in resting-state functional connectivity (Gorgolewski et al., 2014). In this approach, a questionnaire was utilized that prompted for agreeing or disagreeing with given statements. Examples of the statements include presence of social cognition, thinking about present vs. past, and thinking in words vs. images. Subjects rich with imagery showed differential visual-area connectivity (Gorgolewski et al., 2014).

Notably, it seems that there can be differences between online vs. post-scanning experience sampling (Hurlburt et al., 2015). The challenge of sampling subjective experience without affecting the experience itself is well known in studies on consciousness (Block, 2005; Dehaene et al., 2006). How can we know whether one has a conscious experience without resorting to cognitive functions such as attention, memory, or inner speech? Naturally, this is an intrinsic limitation in cognitive science: how to sample experience without altering its neural underpinnings. We discuss this in the following.

## Some Caveats of Self-Reports and Potential Ways to Circumvent Them

Self-report methods have their potential caveats. First of all, it is poorly understood to what extent humans can be self-aware of what is on their minds, due to limits of consciousness. While we may not be fully self-aware, it can be argued that with proper experience-sampling methods a good deal of the inner workings of our minds can be uncovered (Hurlburt et al., 2017). Importantly, self-reports can be used in human research, in contrast to animal models. Thus, further development of experience-sampling methods has the potential of broadening the scope of findings that can be obtained exclusively in human studies. In order to achieve reliable and valid ratings, subjects need to be explained very clearly what they are to rate. For example, inter-rater reliability was reduced when subjects rated goal-directed movements in movie clips compared with presence of faces in them (Lahnakoski et al., 2012).

Another limitation of self-reports is that they take time. For example, obtaining continuous valence and arousal measures for emotional movie clips shown to subjects during fMRI, the clips had to be played twice to the experimental subjects after the fMRI scanning session, one time for assessment of valence, and another time for assessment of arousal (Nummenmaa et al., 2012). In practice, the amount of time that a single subject can be expected to participate in an experiment is limited to a few hours and thus the number of variables that can be sampled is very limited.

The third limitation of self-reports is that they easily alter the experience of movies and narratives if they are obtained during neuroimaging, and on the other hand they can be less reliable if they are obtained separately after scanning (Hurlburt et al., 2015). In practice, however, it seems re-presenting the stimulus after scanning offers a powerful memory cue for retrospective self-reports of how the stimulus was experienced. This has been empirically shown in case of ratings of the humorousness and certain experienced emotions elicited by movie clips (Raz et al., 2012; Jaaskelainen et al., 2016), however, the assumption needs to be validated for other types of self-reported experiences.

There are other types of measures that can be obtained to either replace or complement verbal self-reports. It is possible to ask subjects to paint body maps of feelings that relate to their emotional experience (Nummenmaa et al., 2013). This can be one way to circumvent potential confounds that arise when asking subjects to verbally categorize their emotions. Besides self-reports, emotions can be estimated from physiological data (i.e., galvanic skin response and heart-rate measures; Wang et al., 2018) and facial muscle activity (Jonghwa and Ande, 2008; Jerritta et al., 2014), respectively. Notably, these measures can be recorded simultaneously with fMRI.

Recording of eye-movements provides yet another complementary behavioral measure. In one study, it was observed that variations in eye-movements failed to predict variations in brain activity beyond the early visual areas (Lahnakoski et al., 2014). Other studies have noted that visual representations are relatively independent of eye-movements in hierarchically higher visual areas (Lu et al., 2016; Nishimoto et al., 2017). However, there are findings demonstrating a coupling between eye-movements and hippocampal activity (Hannula and Ranganath, 2009). This suggests that eye-movements have the potential of offering indirect information about the inner workings of the human mind. In the context of eye-movement recordings, variations in pupil size can be taken as an additional measure of arousal (Wang et al., 2018). However, this is easily confounded by constantly changing luminance levels in the case of watching movies, which need to be taken into account. Pupil size has been also linked to processing load, yet on this we failed to obtain significant results in one of our studies (Smirnov et al., 2014). Thus, more studies are needed to determine in which ways eye-movement recordings can be useful in enhancing our understanding of how subjects experience media-based stimuli.

## Neuro-Phenomenology

One potential way to circumvent these caveats and to optimize the synergy between experience sampling and neuroimaging was proposed over two decades ago by Francisco Varela (Varela, 1996; Rodriguez et al., 1999), who coined the term “neuro-phenomenology.” Phenomenology is the philosophical study of the structures of experience and consciousness from the first-person perspective (Callagher and Zahavi, 2020). Varela was inspired by pioneering works of the phenomenologists Merleau-Ponty (1978) and Husserl (1999). Following-up previous attempts to bridge the gap between neural activity of the mind and the subjective experience (e.g., “Hard Problem of Consciousness”) (Chalmers, 1995), Varela reframed the gap by methodologically integrating the two realms, while merging the quantitative statistical power of neuroimaging parameters with the experiential first-person descriptions. However, thus far there has not been any systematic empirical implementation of the neurophenomenological approach (Berkovich-Ohana et al., 2020).

Movies and narratives provide an excellent opportunity to this end: while early neuroimaging tended to rely on dichotomous and over-simplified approaches, the paradigm shift to use media-based stimuli in neuroscience has emphasized the need for a neurophenomenological approach that would accommodate the complex and multifaceted features of natural phenomena. At the theoretical level, phenomenological representations and concepts can accommodate and clarify neural mechanisms and processes, as instantiated in the recent Graded Empathy Framework (Levy and Bader, 2020). This neurophenomenological model of empathy leans on phenomenological analyses revealing parametric leveling of the empathic experience, and these straightforwardly align with neural mechanisms during experiments with movie and narrative stimuli. Accordingly, movies and narratives often evoke multiple levels of neural processes, and phenomenological analyses can describe these processes from a naturalistic and experiential point of view. In that way, neuro-phenomenology introduces a new dimension to the field of neuroscience – this dimension extends self-reported measures. Thus, neuro-phenomenological approaches may further advance neuroimaging research with media-based stimuli.

## Moving Beyond Self-Reports?

Another intriguing, yet completely different possibility is emerging for estimating experienced emotions based on distributed brain activity patterns. Specifically, it seems that it is possible to classify, in many instances well above chance level, basic emotions such as anger, fear, sadness, or happiness from fMRI data using multi-voxel pattern based machine-learning algorithms (Kragel and LaBar, 2015; Saarimäki et al., 2016). Recently, this has been demonstrated also in case of social emotions such as pride and love (Saarimäki et al., 2018). In addition, brain activity accompanying sadness differs between two types of sadness-inducing movies, one involving also sympathy and another involving also hate (Raz et al., 2012). Emotion classification can be accomplished also from EEG data at accuracies as high as 80% (Shuang et al., 2016; Yano and Suyama, 2016; Özerdem and Polat, 2017). EEG also suffices for estimating emotional valence – i.e., negative vs. positive emotional state (Costa et al., 2006; Zhao et al., 2018). Notably, in a recent study, emotional brain activity states were tracked over time during resting state fMRI. The results suggested transitions between emotional states once every 5–15 s during the resting state (Kragel et al., 2021).

Taken together these results suggest that it is possible in principle to assess emotional states that a given subject experienced based on brain activity that is recorded during viewing of movies and listening to narratives. Intriguingly, while at this point rather tentative leads than readily usable tools, these types of approaches might provide ways to circumvent one of the major challenges in cognitive science by allowing one to sample experiences as they take place without altering its neural underpinnings *via* intrusive prompts for self-reports and without the potential loss of validity *via* asking the subjects afterward. These approaches should be further developed and validated in future studies.

## Conclusion

Having detailed annotation of movies and narratives (e.g., presence of people, social interactions, movements) and the recorded brain activity provides only partially the keys for understanding of the neural mechanisms underlying the emotional and cognitive states elicited by the media-based stimuli. To get the complete picture, the inner experiences of experimental subjects need to be additionally estimated. Historically, there have been two opposite schools of thought, one arguing for the usability of introspection (i.e., self-reports) as a method, and the other arguing that introspection is not useful. Several approaches have been developed that can be utilized to tap on different aspects of inner experiences, such as memorization of events, emotional experiences, thoughts, and mental imagery. It seems that post-scanning self-reports are valid for some classes of experiences, such as experiences of humorousness and certain emotions. However, it also seems that for some other inner experiences online-sampling is more accurate. In neuroimaging studies, utilizing movies and narratives as stimuli and applying introspective self-reports can capture essential information that helps guide the neuroimaging data analysis. We foresee that further development of such methods will open new important avenues in cognitive neuroscience. Finally, machine learning approaches that allow for classification and tracking of emotional (and possibly other mental) states based on neuroimaging data may have potential for yielding complementary information to that provided by self-reports in the future.

## Author Contributions

All authors listed have made a substantial, direct, and intellectual contribution to the work, and approved it for publication.

## Conflict of Interest

The authors declare that the research was conducted in the absence of any commercial or financial relationships that could be construed as a potential conflict of interest.

## Publisher’s Note

All claims expressed in this article are solely those of the authors and do not necessarily represent those of their affiliated organizations, or those of the publisher, the editors and the reviewers. Any product that may be evaluated in this article, or claim that may be made by its manufacturer, is not guaranteed or endorsed by the publisher.
